# Predicting 5-year-olds mental health at birth: development and internal validation of a multivariable model using the prospective ELFE birth cohort

**DOI:** 10.1007/s00787-025-02730-9

**Published:** 2025-05-15

**Authors:** Emma Butler, Michelle Spirtos, Linda M. O’ Keeffe, Mary Clarke

**Affiliations:** 1 Department of Population Health, Royal College of Surgeons Ireland, Dublin, Ireland; 2 Department of Occupational Therapy, Trinity College Dublin, Dublin, Ireland; 3 School of Public Health, University College Cork, Cork, Ireland; 4 MRC Integrative Epidemiology Unit and Population Health Sciences, University of Bristol, Bristol, UK; 5 Department of Psychology, School of Population Health & Department of Psychiatry, Royal College of Surgeons Ireland, Dublin, Ireland

**Keywords:** Prediction modelling, Child mental health, Neonatal intensive care unit, Population-level

## Abstract

We developed and internally validated a multivariable model to be used in the perinatal period, to predict 5-year-olds mental health, using the ELFE prospective French multicentre birth cohort (n=9768). Twenty-six candidate predictors were used, spanning pre-pregnancy maternal health, pregnancy-specific-experiences, birth factors and sociodemographic risk (maternal age, education, relationship, migrancy and family income). The Strengths and Difficulties Questionnaire total score at 5-years, dichotomised at the recommended cut-off (16), was the outcome. Least Absolute Shrinkage and Selector Operator followed by bootstrapping was used. High and low-risk was classified by ≥8% risk-threshold score. Stability of the model at population- and individual-level and model performance across groups of interest (sex, sociodemographic risk and neonatal intensive care admissions) was also examined. 10 variables (total number pregnancy-specific experiences, sociodemographic risk, maternal pre-existing hypertension and psychological difficulties, gravidity, maternal mental health problems in a previous pregnancy, smoking and alcohol use in current pregnancy, how labour started and infant sex) with a C-statistic of 0.67; 95%CI (0.64-0.69) predicted mental health. The positive and negative predictive value were 12% & 95.4% respectively, leading to 78.8% of children correctly classified. Model performance was similar across groups of interest but increased for children (born ≥33-weeks-gestation) with neonatal admissions (AUC 0.78; 95%CI (0.69-0.87)). This model is most useful for identifying low-risk children. Applying this model in a tiered preventative intervention framework could be beneficial with those predicted to be high-risk receiving further screening to determine the level of intervention required. External validation and implementation research are required before considering its use in practice.

**Supplementary Information:**

The online version contains supplementary material available at 10.1007/s00787-025-02730-9.

## Background

One in six children between the ages of 2 and 8-years has a diagnosable mental, behavioural or developmental disorder [1], more than half of whom are severely affected [2]. They are the predominant chronic diseases of youth, accounting for greater lifespan morbidity and mortality than physical diseases and are the leading cause of lost-life years[3–6]. Preventing or delaying the onset of poor mental health in childhood is a key avenue for improving public health.

Poor mental health is likely to be the result of multiple accumulated risks across time and contexts [7]. When several risk factors occur simultaneously, the prevalence of mental health problems increases markedly [8]. Family socioeconomic status has repeatedly been linked to mental health problems in children [9]. But, socioeconomic risks tend to co-occur, rarely exist in isolation [10] and no single measure has been found to reflect socioeconomic position adequately [11]. Aggregating multiple risk factors can more accurately ascertain the severity of socioeconomic adversity and has more predictive power than focusing on the effects of any single indicator [12, 13]. Prior research has shown that pregnant women experiencing more sociodemographic risks have higher indicators of physiological strain also [14].

Investigating the risks present before a baby is born is a growing field of inquiry, and factors such as smoking and alcohol consumption in pregnancy, maternal mental and physical health, as well as intimate partner violence have been investigated [15–20]. A comprehensive review highlights accumulating evidence that exposure to prenatal stressors increases the risk for later mental health difficulties in the offspring [21]. A variety of postnatal factors, such as maternal mental health, child temperament, family environment, and socioeconomic status also play a part [22, 23]. A life‐course maternal “health-capital” perspective that considers all gains and losses in biological, psychological, and physical health over the lifetime is likely to provide the fullest picture of the impact of environmental exposures [24]. Our previous work demonstrated a dose–response relationship in the association between cumulative pregnancy complications and cumulative sociodemographic risk at birth and child mental health at 5 and 9-years in an Irish longitudinal cohort [25].

How multiple risks, as early as in pregnancy, can *predict* childhood mental health remains less well understood.

Prognostic factors may be causal or non-causal in nature [26]. The National Institute of Mental Health Research Domain Criteria framework aims to understand mental health in terms of dysfunction in biopsychological systems. In 2019, sensorimotor was added as a domain of interest due to its known transdiagnostic feature across mental health presentations [27], but it is unknown whether sensory characteristics are prognostic of later mental health. Developmentally sensorimotor difficulties would be evident long before mental health symptoms and thus has the potential to be a valuable prognostic indicator in the clinical setting.

Prediction models are used to calculate an individual’s estimated risk of an outcome and they guide clinical decision-making by informing the probability of that outcome [28]. No existing prediction model for child mental health can be recommended for clinical practice due to insufficient sample sizes, unrepresentative samples and lack of appropriate testing of model performance (calibration) [29]. These mental health risk calculators are designed for diagnosis specific outcomes such as depression, bipolar and autism and typically do not include extrinsic, contextual factors [30–34]. The discriminative ability of current models typically lies in the 0.6–0.7 area-under-the-curve range [30, 32, 35]. A large gap in research is that none of the 100 reported models [29] were designed to be applied in the perinatal period in the general population. The aims of this study were to: (1) develop and internally validate a multivariable prediction model for mental health at 5-years, using information known in the perinatal period, (2) examine the model’s stability at population and individual-level, (3) examine the performance of the model at differing risk-thresholds and in subgroups of interest, and (4) examine whether the infant’s sensory profile at 1-year is selected as a prognostic factor.

## Methods

Data was obtained from Etude Longitudinale Francaise depuis l’Enfance (ELFE), a prospective French birth-cohort study. Details of the design, sample and measures used are presented elsewhere [36]. The objective of ELFE is to study determinants of child development, health and socialisation from birth to adulthood. Nationwide, 349 maternity units were randomly selected, and babies were recruited from the 320 maternity units that agreed to take part during 25 selected days in 2011. Inclusion criteria were single/twin live births at ≥ 33-week’s gestation, mother ≥ 18-years old, no plan to leave France within 3-years and informed consent signed by the parents or the mother alone, with the father informed of his right to deny consent. More than 96% of the mothers who satisfied the first two inclusion criteria (n = 37,494) were contacted by the ELFE team during their stay in the maternity unit and 51% (n = 18,040) agreed to participate. The women gave birth to 18,329 babies, including 289 twin-pairs. Children were followed-up at 2-months, 1-, 2-, 5- and 10-years and is on-going. Data request from https://plateforme-acces-donnees-elfe-france.site.ined.fr/. This study uses information gathered at birth, 2-months, 1 and 5-years-old. This study was approved by the Research Ethics committee for the Royal College of Surgeons Ireland (RCSI RIMS 212610659).

## Measures

### Candidate predictors

Maternal biological, psychological and social factors from pre-pregnancy to the end of the perinatal period (conception to one year after giving birth) [37] were considered as candidate predictors. These were all self-reported on questionnaires or retrieved from the medical file by the research team during the perinatal period. They were registered a priori on osf.io. https://doi.org/10.17605/OSF.IO/5426Q and with consideration to the sample size calculation (S.1). In brief, they included (1) maternal pre-pregnancy health: gravidity, mental health, history of blood pressure problems or diabetes (outside of or during a previous pregnancy), (2) total pregnancy-specific experiences (see below), (3) smoking and drinking alcohol in pregnancy (binary yes/no), (4) birth/delivery factors incorporating how labour started, mode of delivery, gestational age, neonatal intensive care unit and infant sex and (5) post-natal cumulative sociodemographic risk (see below). (Table 1 and S.2).

**Table 1:** Summary of descriptive characteristics (*n* = 9768)/candidate predictors by outcome group

	All (*n* = 9768) %	Adequate mental health (*n* = 9173) %	Poor mental health (*n* = 595) %
^a^Sociodemographic risk:			
None	31.5	32.6	17.6
Low	29.5	29.7	26.8
Moderate	32.2	31.2	43.9
High	6.8	6.5	11.7
^b^Total# pregnancy-specific experiences:			
0	34.4	35.0	25.7
1	33.5	33.8	29.6
2	18.4	18.0	23.8
3	8.7	8.5	11.2
4 +	5.0	4.6	9.8
Child sex:			
Male	50.1	49.2	62.1
Maternal pre-conception health:			
Psychological problems before pregnant (% yes)	23.1	22.6	30.0
History of blood pressure problems (% yes):			
Outside a pregnancy	1.3	1.1	3.4
Previous pregnancy	1.4	1.4	1.4
History of diabetes problems (% yes):			
Outside pregnancy	1.0	1.0	0.7
Previous pregnancy	2.6	2.6	3.3
Mental health difficulties (depression/anxiety) in a previous pregnancy:			
Never pregnant %	29.2	28.8	33.9
% yes	8.4	7.9	14.7
Maternal health behaviours in current pregnancy:			
Smoked (% yes)	19.5	18.8	29.2
Drank alcohol (% yes)	24.0	23.9	24.7
Birth factors:			
How labour started (% yes):			
Spontaneous	71.9	72.0	71.4
Induced	19.0	18.7	21.9
C-section	9.1	9.3	6.7
Mode of delivery (% yes):			
Normal vaginal delivery	71.0	71.3	66.9
Assisted	11.5	11.2	15.1
Planned C-section	7.1	7.3	5.1
Emergency C-section	10.4	10.2	12.9
Neonatal Intensive Care Unit (NICU):			
% yes	5.2	5.0	7.3
Gestational Age ^d^(weeks):			
Mean (SD)	39.2 (1.4)	39.2 (1.4)	39.1 (1.4)
^c^Sensory-profile @ 1:			
Typical Sensory	80.4	81.4	67.5
Possible sensory difficulties	6.5	6.6	5.4
Definite sensory difficulties	13.1	12.0	27.1

^a^Sociodemographic risk comprised of maternal age, education level, relationship status, migrancy and family income quintile. ^b^Pregnancy Specific Experiences comprised of biological factors (bleeding, high blood pressure/diabetes during pregnancy and infertility treatment), psychological factors (prefer not to be pregnant, persistent psychological difficulties, recurrent miscarriage, difficult pregnancy) and social factors (father not at delivery, not having enough support, over-crowding and inadequate living conditions) that specifically occurred during pregnancy. ^c^Sensory Processing group when child was one-year old derived from a latent class analysis of ten behavioural indicators. ^d^Children born < 33-weeks-gestation were not included in the ELFE study

### Total pregnancy-specific experiences (PSEs)

We previously demonstrated a dose–response relationship between cumulative biological pregnancy complications and child mental health [25]. Expanding this further, the PSEs variable used here was derived from summing the following items that were either self-reported by the mother on a questionnaire or recorded in the medical chart: biological factors (bleeding, high blood pressure/diabetes during pregnancy and infertility treatment), psychological factors (prefer not to be pregnant, persistent psychological difficulties, recurrent miscarriage, difficult pregnancy) and social factors (father not at delivery, not having enough support, over-crowding and inadequate living conditions) that specifically occurred during this pregnancy, with a maximum possible count of 12. This ranged from 0–4 + (S.2, Fig. S.1 & T.1).

### Post-natal cumulative sociodemographic risk (SR)

A sociodemographic risk (SR) score, developed and validated previously in pregnant women [14], incorporating maternal age, migrancy, family income quintile, maternal education and relationship status when the child was 2-months old was constructed. Both higher (36 + years) and lower maternal age at birth (15–25 years) were considered risks. Migrancy was dichotomised with “mothers’ birth outside of France” considered a risk. Family income was summed from all sources, after tax and social insurance and divided by the number of people in the household. A 3-category variable was created from quintiles to denote high (Q4&5), middle (Q3) and low income (Q1&2). Maternal education had 4 levels: 1) no formal education, up to and including any level of secondary school education, (2) post-secondary training up to and including diploma, (3) degree-level and (4) post-graduate (MSc/PhD) with less education corresponding to higher risk scores. Lastly, being married/living with a partner was considered low risk, and single/widowed/divorced/separated/never married considered higher risk. Values were then added and divided into four sociodemographic risk quartiles: none, low, moderate and high. These SR categories aligned with primary caregiver’s response to the question “Currently for the household would you say that financially you are: comfortable, OK, tight, difficult, can’t get by without going into debt, does not wish to answer?” also measured at 2-months. For example, respondents who reported “always in debt” had the highest proportion in “high SR”, respondents who reported “financially comfortable” had the highest proportion in “no SR” (S.3).

### Sensory processing

To address aim 4, the child’s sensory profile group at 1-year was added as a possible candidate predictor. This sensory profile was derived from a Latent Class Analysis of behavioural indicators, identified by a public and patient involvement group as reflecting sensory processing, reported by the caregiver when the child was 1-year-old, please see Butler et al. (2025) for further details [38]. It is comprised of three latent sensory classes: ‘typical sensory’, ‘possible’ and ‘definite’ sensory difficulties. (S.4.)

### Outcome measurement

The strengths and difficulties questionnaire (SDQ) was completed by the caregiver when the child was 5-years-old, as a measure of child mental health & well-being. The SDQ is a valid and reliable instrument to screen for emotional and behavioural problems in children aged 3–16 years and is widely used in research and clinical practice [39]. The SDQ is a parent-rated questionnaire containing 25-items on a 3-point likert scale (0 = not true; 1 = somewhat true; 2 = certainly true), five items are reverse scored. Item scores are aggregated into 5 subscales. The first four subscales combine to calculate a total score ranging from 0–40. Higher scores indicate higher difficulties. The SDQ-total was dichotomised at the recommended cut-offs (sdqinfo.org). Total scores of 17 or above were considered to be in the clinical range. Parent-reported SDQ-total scale has higher internal consistency (Cronbach’s-alpha = 0.82) and test–retest reliability than the four subscales [40]. The SDQ has been validated in French samples [41] and the total-score has been demonstrated to distinguish between clinic and community French samples [42].

#### Participants in the analysis

Only participants who participated in the 5-year wave and thus had the outcome available (*n* = 11,248) and had complete information on the candidate predictors (*n* = 9768) were included in this analysis.

### Data analysis

We constructed two of the predictor variables as described above. As we only had one continuous predictor (gestational age in weeks), non-linear relationships (such as fractional polynomials) between this and the outcome were examined. The univariate associations between each categorical candidate predictor and the outcome was explored (S.5). This information was not used for variable selection, rather, that was achieved using Least Absolute Shrinkage and Selector Operator (LASSO). Bootstrapping (1000 repetitions) was used to penalise for the known optimism of prediction models in development data as a result of over-fitting to estimate optimism-corrected performance (internal validation). As the outcome was binary, the models were developed using logistic regression. We inputted < 33 **candidate** predictors as per the sample size calculation (S.1). Fig S.2 shows the mathematical equation for calculating each individual’s risk score. For aim 4, we repeated the above process but added the sensory variable as a candidate predictor.

Model predicted probabilities were compared with actual outcomes using several performance metrics and related plots: C-statistic (AUROC) which distinguishes between those with and without mental health problems and ranges from 0–1. Calibration measures how well the predicted outcome of the model agrees with the observed outcome on average. A perfectly calibrated model has a slope = 1. Slope < 1 indicates over- whilst slope > 1 indicates under-fitted respectively [43]. For calibration-in-the-large (CITL), CITL < 0 indicates predictions are too high. Brier score ranges from 0–25 with lower values indicating better fit.

#### Stability of the prediction model

Stability in estimated risk is arguably one of the most important aspects to consider when developing a model and in the absence of further validation, the quantification of instability is essential as it exposes the uncertainty and fragility of new models [44]. Therefore, we examined the following instability plots: prediction, calibration and classification (using ≥ 8% threshold). We also calculated the average mean absolute prediction error (MAPE).

##### Thresholds

A traditional method for operationalising clinical utility is by evaluating positive predictive value (PPV) and negative predictive value (NPV) [45]. Sensitivity, specificity, PPV and NPV were calculated (Fig. S.3) at multiple risk-threshold points (T.3) as no standard criteria for identifying a risk-threshold exist for the prediction of childhood mental health. Sensitivity relates to how well the model can classify children who truly have poor mental health. PPV, on the other hand is the proportion of children predicted to be high-risk that had poor mental health at 5-years [46]. Whilst PPV is the usual test of performance, it can inaccurately reflect test performance when the overall base prevalence is low [46].

##### Subgroups

Prediction models need to be evaluated for important subgroups to help ensure fairness and accuracy in under-represented groups [47]. We compared whether there was evidence of differential performance across groups of interest (sex, sociodemographic risk and neonatal intensive care unit (NICU)) by examining the models performance using the ‘roccomp’ function in STATA [48].

##### Missing data

We examined characteristics of participants included in our model versus those excluded from the analysis due to missing predictor or outcome data (S.6a). We also examined the distribution of missing predictor data (S6.b&c).

Due to the complex survey design of the data, models included a cluster variable (maternity unit) and sampling weights. The clustering variable accounted for nested observations of babies within hospitals and adjusted the standard error accordingly. Statistical analysis was performed with STATA v.17 [48].

## Results

Of the *n* = 9768 participants, *n* = 595 (6.1%) had the outcome of interest i.e. poor mental health. Baseline characteristics are summarised in Table 1. Almost two-thirds of the sample experienced none to low sociodemographic risk and none or one total pregnancy-specific-experience. 50% of the babies were male and the majority were delivered via spontaneous normal vaginal delivery. The mean gestational age was 39.2 weeks (minimum gestational-age in weeks was 33-weeks) and 5.2% were transferred to a NICU.

Participants excluded from analysis (Fig. S.4) due to loss to follow-up (*n* = 7081) or missing some candidate predictors (*n* = 1480) had slightly higher levels of: cumulative sociodemographic risk, maternal history of psychological difficulties and planned C-sections and marginally lower gestational-age. No other variables were substantially different (S.6a).

The prediction model for the risk of poor mental health in childhood is presented in Table 2. Ten predictors were selected in the LASSO model: total number pregnancy-specific-experiences, cumulative sociodemographic risk, maternal pre-existing hypertension and maternal pre-existing psychological difficulties, gravidity, maternal mental health problems in a previous pregnancy, smoking and alcohol use in current pregnancy, how labour started and infant sex. Discrimination (AUCs) did not change significantly after internal validation using bootstrapping i.e. optimism-adjusted (AUC 0.66 (95%CIs: 0.57–0.71) (S.7). Calibration was good as evidenced by the calibration slope (1.04) and gradient. The bootstrap uniform shrinkage factor is −0.02 (S.7). The slope indicates that for low probabilities, our predictions are not low enough and for high probabilities, predictions are not high enough.

**Table 2:** Model performance and intercept and coefficients for the LASSO prediction model for childhood mental health (SDQ-total > 16) in children aged 5-years (n = 9768)

Predictors	^a^Model Coefficient
Intercept	−2.80
^b^Total number of pregnancy-specific experiences:	
0	Ref
1	.07
2	.51
3	.56
4 +	1.24
^c^Sociodemographic risk:	
None	Ref
Low	.15
Moderate	.59
High	.71
History of pre-morbid blood pressure (not during a pregnancy):	
No	Ref
Yes	.21
History of psychological difficulties before being pregnant:	
Yes	Ref
No	−0.12
Mental health difficulties in a previous pregnancy:	
Yes	Ref
No	−0.39
Never pregnant before	.03
Smoked during the pregnancy:	
Yes	Ref
No	−0.35
Consumed alcohol during the pregnancy:	
Yes	Ref
No	-.002
How labour started:	
C-section (plan/emergency)	Ref
Spontaneous	−0.03
Induced	0.07
Infant sex:	
Female	Ref
Male	0.40
Discrimination & calibration performance metrics:	
Area Under Curve (AUC)	0.67 (0.64–0.69)
Calibration slope	1.04
Expected/Observed Ratio	1.03
Calibration-in-the-large (CITL)	−0.03
Brier Score	0.06

^a^coefficient from LASSO ie includes shrinkage. ^b^Pregnancy Specific Experiences comprised of biological factors (bleeding, high blood pressure/diabetes during pregnancy and infertility treatment), psychological factors (prefer not to be pregnant, persistent psychological difficulties, recurrent miscarriage, difficult pregnancy) and social factors (father not at delivery, not having enough support, over-crowding and inadequate living conditions) that specifically occurred during pregnancy. ^c^Sociodemographic risk comprised of maternal age, education level, relationship status, migrancy and family income quintile

### Stability

**Fig. 1:**
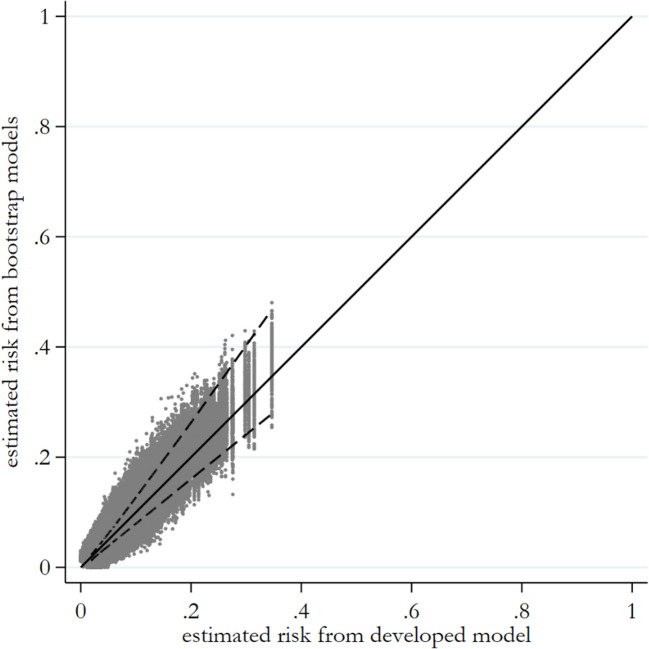
Prediction instability plot. Note: scatter of each individuals bootstrapped predicted values (Y-axis) against their predicted value from the original model (X-axis) and their 95% range

Our model shows good stability at population and individual-level across metrics. The prediction instability plot (Fig. 1) is a scatter of each individuals bootstrapped predicted values (Y-axis) against their predicted value from the original model (X-axis) and their 95% range. Our plot shows that, for example, for a child with an original estimated predicted risk of 0.3, their bootstrapped 95% range of estimated risk is between 0.22–0.41. Defining our risk-threshold for high-risk at 0.08 (see thresholds below), their 95% range of predicted estimates are all within the high-risk range.

For the calibration instability plot (Fig. S5a), the bootstrapped curves are overlaid on the same plot together with the calibration curve from the original model. The wider the spread of the bootstrapped curves, the greater concern for instability. Our calibration curves begin to deviate from the 45-degree line once risk exceeds 0.2. (as our risk-threshold is 0.08, all those categorised as high-risk will still be high-risk). This variability may still be reliable as they are still categorised the same overall. There is much less variability when risk is < 0.2.

The classification plot (Fig. S.5b) is a scatterplot of each individuals classification index (Y-axis) plotted against their original predicted classification (X-axis). Our plot has a very narrow distribution with individuals values close to zero for most children except those with predictions close to the risk-threshold (0.08).

In terms of discrimination, the C-statistic instability histogram (Fig. S5c) shows very little variability in C-statistic estimates across bootstrapped samples (all between 0.65–0.66). Furthermore, our average MAPE is 0.006. This means that, on average, across children, the absolute difference between their developed model prediction and the bootstrapped model’s prediction is 0.006, indicating instability in each individuals prediction is very low (Fig. S5d).

### Thresholds

The percentage of children identified as at-risk of poor mental health for different risk-thresholds are presented in Table 3. As there is no agreed cut-off in the literature on what constitutes ‘high-risk’ of future poor mental health, sensitivity, specificity, PPV and NPV was calculated at risk-thresholds of 8,15, 20 and 25%.

**Table 3:** The categorisation of children as predicted ‘high-’ or ‘low-risk’ of poor mental health (SDQ-total > 16) at 5-years based on the prediction model using different levels of risk-thresholds compared with their observed outcome

Proportion identified at high-risk % (*n*)	Predicted ‘low-risk’ group % (*n*)		Predicted ‘high-risk’ group % (*n*)	
	Observed Adequate mental health	Observed Poor mental health	Observed Adequate mental health	Observed Poor mental health
@25% risk-threshold				
	*N* = *9725*		*N* = *43*	
0.4 (43)	94.0 (9142)	6.0 (583)	72.1 (31)	27.9 (12)
@20% risk-threshold				
	*N* = *9644*		*N* = *124*	
1.3 (124)	94.1 (9073)	5.9 (571)	80.7 (100)	19.3 (24)
@15% risk-threshold				
	*N* = *9427*		*N* = *341*	
3.5 (341)	94.3 (8892)	5.7 (535)	82.4 (281)	17.6 (60)
@8% risk-threshold				
	*N* = *7820*		*N* = *1948*	
20.0 (1948)	95.4 (7459)	4.6 (361)	88.0 (1714)	12.0 (234)

Using a 15% risk-threshold, the model identifies 3.5% of children at-risk, with a sensitivity of 10.1%, specificity of 96.9%, PPV of 17.5% and NPV of 94.3% (T.3). As we reduce the risk-threshold to 8%, the model identifies 20% of children at-risk, sensitivity increases (39%), specificity decreases (81.3%), PPV decreases (12%) and NPV increases (95.4%).

Whilst the 25% risk-threshold had the best PPV (27.9%), in terms of absolute numbers, the 8% risk-threshold (Fig S.6) had the highest sensitivity, that is, the highest number of children classified as being high-risk who actually had poor mental health at 5-years, *n* = 234/595 (39%).

### Subgroups

**Fig. 2:**
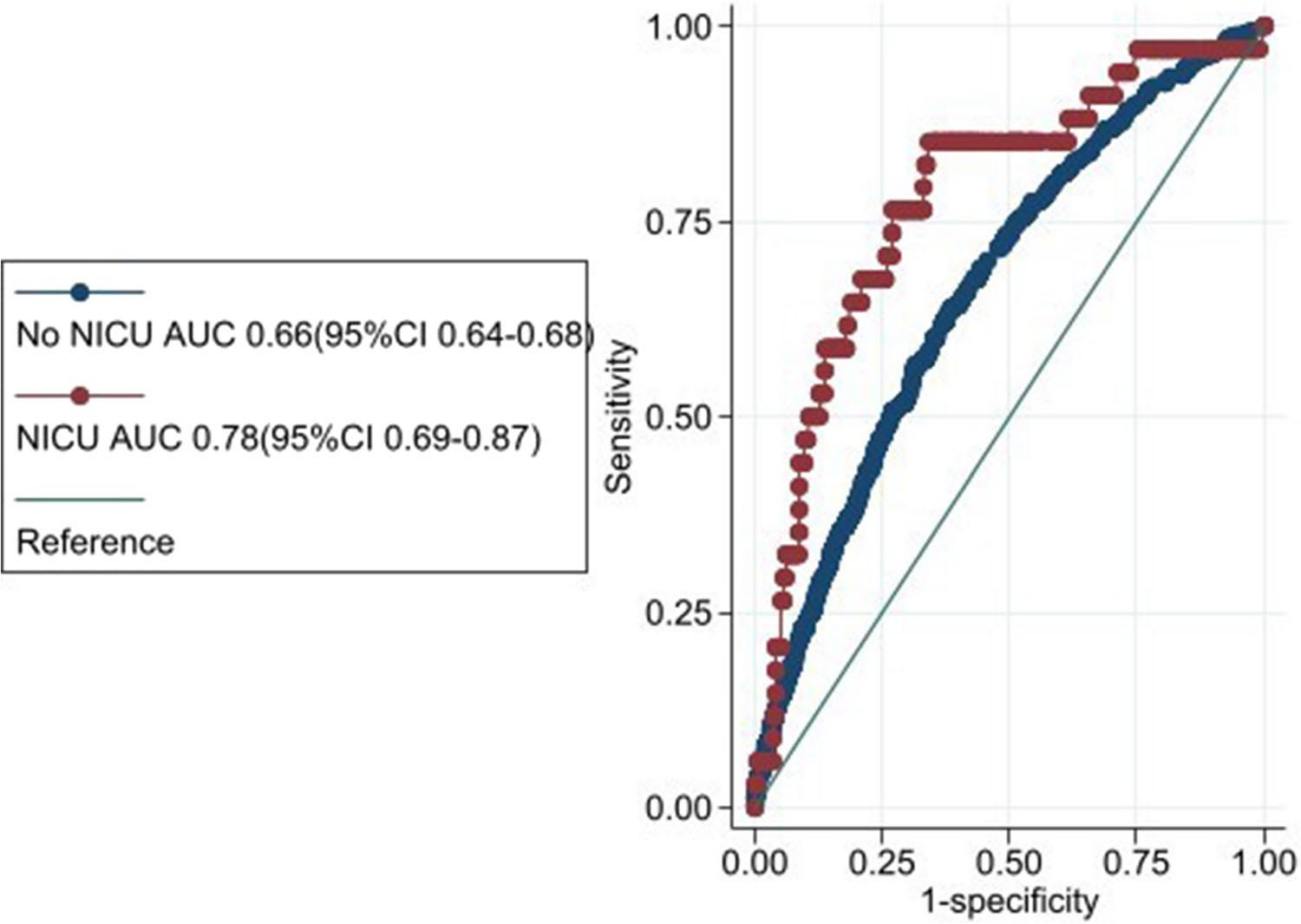
Receiver Operating Curve (ROC) plot comparing the model’s performance in children who did & did not spend time in neonatal intensive care unit (NICU). Note: optimism-adjusted 95%CI’s do not overlap

We examined the prevalence of poor mental health across our groups of interest (S.8). Males had worse mental health (8% to 5% females). The known dose–response between sociodemographic risk groups and the proportion with poor mental health was evident (4%, 6%, 9% and 13% for none, low, moderate and high sociodemographic risk respectively). There was no significant difference in the proportion of children with poor mental health between those who did (7%) and did not (6%) spend time in the NICU (S.8). There was no significant difference in the model’s performance (AUC) between males and females or between levels of sociodemographic risk (Table S.9). However, the model was significantly more accurate for children (born ≥ 33-weeks-gestation) who reported being in NICU. AUC 0.66 (95%CI 0.64–0.68) and AUC 0.78 (95%CI 0.69–0.87) for non-NICU and NICU respectively. (Fig. 2 & S.9).

Lastly, for our final aim, we included the child’s sensory profile at 1-year as a candidate predictor. The LASSO selected the sensory variable as a predictor in the model (co-efficients shown in S.10) showing that sensory profile could be considered as a possible prognostic factor in future studies. However, the addition of the sensory information at 1-year to the model did not significantly improve the model’s performance compared to the model at birth without the sensory information. AUC 0.69 (95% CI 0.67–0.71) and AUC 0.67 (95% CI 0.64–0.69) for sensory and birth model respectively (S.10).

## Discussion

Our analysis shows that it is possible to predict poor mental health at 5-years using information known in the perinatal period with fair to moderate accuracy. We developed and internally validated a prediction model to be used in the perinatal period with a view to providing early intervention in the first 1001 days. Established risk factors for poor mental health that were included in the identified prediction equation were sociodemographic risk, infant sex, history of maternal psychological difficulties (in and outside of a pregnancy), smoking and alcohol use in pregnancy. The model also included total number of pregnancy-specific-experiences for which there are mixed findings in the literature [49–52]. Novel predictors included pre-existing maternal hypertension (over and above high blood pressure during a pregnancy), how labour started and the child’s sensory profile at 1-year. Whilst the addition of the sensory information did not improve the overall model performance, future studies should include a standardised measures of sensory processing in order to examine whether a standardised sensory measure improves model performance, but this is currently unavailable in longitudinal population cohorts.

Our multiple (in)stability assessments (prediction, calibration, classification and MAPE) provide reassurance that the prediction model is likely to be reliable at both population- and individual-level, and overcomes some of the criticisms of existing models [29] however, further validation of the model (in an external validation study) is still required.

As the outcome is rare (6.1%), this model performs best for stratifying children who *do not need* to be referred to early intervention, that is, the NPV rate. This is a known phenomenon with rare outcomes, that is, the more certainty there is that a negative result indicates absence of outcome but with less certainty that a positive test really indicates outcome presence [53, 54]. Of the children predicted to have a low-risk of poor mental health, 95.4% of them do not have mental health difficulties at 5-years. We are less confident in its ability to identify the children who *definitely need* early intervention, as the PPV is lower i.e. increased false positives. Of the 1,948 predicted to be high-risk, only 234 actually have poor mental health by 5-years (12%). Because of the low PPVs, our model should not be considered a ‘gold standard’ and improving the model’s performance for predicting mental health in childhood remains an important aim for future research. Depending on the intended use of the model, this may involve the development of a new prediction model to achieve better positive prediction. This is necessary if for example, the intended use of the model was to offer high-risk children an expensive or invasive intervention. On the other hand, this model has excellent NPV. This is useful if the intention is to identify babies who likely do not require additional support/early infant mental health intervention i.e. screen-out children. The predicted high-risk group could then be offered more in-depth screening or assessment to determine their level of intervention required e.g. parent education, family therapy etc. rather than adopting a “watch and wait” approach [55]. Additionally agreeing differing risk-thresholds with a view to applying a tiered model of intervention delivery [56] is a possible solution to reduce the system impact of false positives, as the level of the risk-threshold impacts the sensitivity, specificity, PPV and NPV (T.3). A tiered-model as opposed to a binary cut-off would involve the provision of universal supports for low-risk children, targeted intervention for the moderate-risk group (for example, referral to clinician for further assessment) and intensive intervention services for an ultra-high-risk group [56].

Risk prediction models are used to inform individualised decisions about treatment/monitoring therefore it is important they are robust and reliable for all users [44], this is particularly important in the intended population-level use. We assessed model performance across sex, sociodemographic risk and NICU given that under-estimating or over-estimating risk for some groups could potentially lead to reduced access to preventative care or unfair burden respectively. Reassuringly, our model performance was similar across sex and sociodemographic risk groups. It was particularly good for children (born ≥ 33-weeks-gestation) who had been in a NICU and thus this cohort may be a population for whom this model may be particularly useful.

The NICE quality statement on promoting health and well-being in under 5’s (National Institute for Health and Care Excellence, 2016) recommends that parents of children less than 5-years-old have a discussion during each of the 5 key contact developmental checks about factors that may pose a risk to their child’s social and emotional well-being. They do not specify how to measure this and/or what thresholds of risk are acceptable. We have presented information here on a range of differing risk-thresholds, as a challenge is the lack of consensus on the optimal ethical and mathematical criteria for algorithmic fairness, which needs to take the relative costs of false positives (eg increased patient burden and potential for stigma) and false negatives (eg reduced access to intervention) into account [57]. Further work should include seeking agreement from stakeholders on different risk-thresholds and the economic and service implications of such risk-thresholds.

Identifying high-risk dyads/families at the earliest timepoint, such as during routine developmental health checks in community care, would enable intervention to support both infant mental health and family-centred care before mental health difficulties are evident. Whilst this model shows considerable promise, external validation as well as testing the feasibility, acceptability and usability of it, are next steps.

### Strengths & limitations

The use of a population-based sample for model development is a key strength which enhances generalisability. Calculating an appropriate sample size a priori reduced the likelihood of over-fitting. Using appropriate statistical methods, for example, investigating whether fractional polynomials were required and correcting for optimism, in the modelling process adds to the robustness. Adjusting for clustering is also a strength and providing (in)stability metrics provides reassurance that the model is reliable. The data used here is from a middle-high resource setting and so the performance of the model using data from low-resource settings requires testing to determine its generalisability.

Neonatal intensive care and gestational age in weeks were not selected as predictors using LASSO. This could be due to ELFE only recruiting children ≥ 33-weekgestation. As a result, this model cannot be applied to children born < 33-weeks-gestation. However, its performance accuracy for children who were in NICU & ≥ 33-weeks (ie moderately-late preterms) is very good (AUC 0.78).

Despite a low level of missing data, restricting to complete-case analysis rather than carrying out multiple imputation is a potential limitation. However, using incorrectly specified models for multiple imputation would arguably increase bias.

The sensory group metric used was a proxy representation of the child’s sensory profile at 1-year in the absence of the availability of validated sensory measures in population-level datasets. Future research could develop this avenue further by applying the method with a standardised, validated sensory measure. Longitudinal cohorts should consider adding a sensory outcome measure to enable furthering this line of inquiry.

The positive predictive value of this prediction model is limited, and its strength lies with the intended use of screening-out children at population-level who have a predicted low-risk of later poor mental health. However, predictive values are correlated with base rates and it has been argued that relying on these frequentist metrics may under-estimate the utility of algorithms for outcomes with low base rates as is the case here [54].

## Conclusions

Our work shows that it is possible to predict poor mental health at 5-years using information known in the perinatal period. Our model showed good classification performance and calibration particularly for children (born ≥ 33-weeks-gestation) who spent time in NICU. This prediction model was generated using only non-invasive, low-cost information making it easier to implement in clinical practice as it incorporates information that can be self-reported and aligns with routine developmental check appointments in the early years.

## Supplementary Information

Below is the link to the electronic supplementary material.

Supplementary file1 (DOCX 257 KB)

## Data Availability

Data request from https://plateforme-acces-donnees-elfe-france.site.ined.fr/
