# Supplementary material for: Predicting 5-year-olds mental health at birth: development and internal validation of a multivariable model using the prospective ELFE birth cohort
Source: Eur Child Adolesc Psychiatry. 2025 May 15;34(10):3185–96. doi: 10.1007/s00787-025-02730-9 (PMC12592302; doi:10.1007/s00787-025-02730-9)
Supplement: Supplementary file 1 — Supplementary file1 (DOCX 257 KB) [file 787_2025_2730_MOESM1_ESM.docx]

**Table S.1:**

***Sample size calculation a priori based on full sample at 5-years (n=11248) and then () based on participants included in analysis (n=9768).***

Shrinkage and over-fitting is much less of a concern if the sample size is appropriate for the number of predictors and events/participants with the outcome of interest. For our binary outcome at 5-years, we have 697/11248 (595/9768) with the event. Therefore, the following sample size calculations apply:

| Assumed C-statistic | Inputs | EPP |
| --- | --- | --- |
| 0.84 | 135 (114) | 5.17 (5.14) |
| 0.74 | 59 (50) | 11.82 (11.72) |
| 0.70 | 40 (33) | 17.43 (17.76) |
| 0.64 | 19 (16) | 36.7 (36.6) |

Aiming for a modest C-statistic (0.70), we input a maximum of 33 ***candidate*** predictors. It is important to note that each *level* of a factor variable is considered one input.

| **Table S.2: list of all pre-conception, perinatal or birth characteristics considered as candidate predictors:** | | |
| --- | --- | --- |
| Variable name | Timepoint Measured &  Respondent | Question used/code |
| **Sociodemographic risk** | | |
| Maternal Age at birth (years)  Mean (SD) | In maternity unit at childs birth, Mother self-report |  |
| Mother born in France  (No) | In maternity unit at childs birth, Mother self-report | What is your nationality? French acquired, foreign and stateless coded as No, French born coded as yes |
| Maternal Education Level | Survey when child was 2 months old, Mother self-report | M_educ@2m: based on ISCED, none, primaire and secondaire coded as 3, lycee coded as 2, bac+2 coded as 1 and >bac+2 coded as 0. (higher code equates with less education). |
| Postgrad |  |  |
| Degree |  |  |
| Leaving Cert |  |  |
| None up to & incl J. Cert |  |  |
| Family income | Survey when child was 2 months old, Mother self-report | Taking account of all types of income in the household what is current amount of net monthly resources? ELFE Team made derived variable of equivalised income quintiles. |
| Highest two quintiles |  |  |
| Middle quintile |  |  |
| Lowest two quintiles |  |  |
| Mother in a relationship at birth | In maternity unit at childs birth, Mother self-report | Are you currently in a couple? (yes/no) |
| Cumulative Sociodemographic Risk (SR) |  | Constructed variable from summing maternal age & education level, relationship, migrancy and family income. |
| Each pregnancy specific experience | | |
| **Biological:** |  |  |
| Late bleed (2^nd^/3^rd^ trimester) in this pregnancy | In maternity unit at childs birth, Medical file | No, placenta previa, retroplacental haematoma, other (all responses except NO considered as YES) |
| High blood pressure (HBP) in this pregnancy | In maternity unit at childs birth, Medical file | HBP developed during pregnancy (systolic ≥140mmHg or diastolic ≤90mmHg): No, yes with proteinuria, yes without proteinuria (all responses except NO considered as YES) |
| Gestational Diabetes Mellitus (GDM) in this pregnancy | In maternity unit at childs birth, Medical file | Gestational Diabetes: no, yes, Don’t Know coded as no. |
| Did you have infertility treatment for this pregnancy? | In maternity unit at childs birth, mother self report | yes/no |
| **Psychological:** |  |  |
| Prefer not to be pregnant/would have liked it later | In maternity unit at childs birth, Mother self-report | When you found out you were pregnant: happy, liked it earlier, (coded NO) liked it later, prefer not to be pregnant (coded YES) |
| Persistent psychological difficulties in this pregnancy | In maternity unit at childs birth, Mother self-report | During the pregnancy did you have any persistent psychological difficulties? Yes/no |
| >1 previous miscarriages | Survey at 2 months old, Mother self-report | How many miscarriages have you had before this pregnancy? #coded as >1 (yes) v 0/1 (no) |
| Difficult pregnancy | Survey at 2 months old, Mother self-report | How would you describe your pregnancy? A pleasant time, fairly pleasant, (coded NO) a difficult time (coded YES) |
| **Social:** |  |  |
| Father **not at** delivery | In maternity unit at childs birth, Mother self-report | Was the father present at the delivery? yes/no |
| Not enough support | Survey at 2 months old, Mother self-report | If had (no) partner – during your pregnancy do you think (people close to you) your partner supported you: very well, well, not much, not at  all (not much & not much at all coded as not enough support, rest coded as enough support) |
| >5 people in house | Survey at 2 months old, Mother self-report | How many people generally live in your household? Number given was recoded as >/<5. |
| Criticisms about home | Survey at 2 months old, Mother self-report | Do the following criticisms apply to your dwelling?: too Small, too damp, mould on walls, noisy (internal/external) – counted #yes/4 |
| Total number pregnancy-specific-experiences (total of previous 12 endorsed). |  | Range 0-4+ |
| **Maternal health behaviours in pregnancy:** | | |
| Smoked in pregnancy | In maternity unit at childs birth, Mother self-report | Did you smoke during your pregnancy even just from time to time? Yes/No/(Don’t Know coded as No) |
| Alcohol in pregnancy | In maternity unit at childs birth, Mother self-report | During your pregnancy how often did you consume alcoholic drinks? Never (coded no), once/month or special occasions, 2-4times per month, 2-3times per week, 4+times/week but not daily, everyday, only before knew pregnant, prefer not to answer (all got 1 indicating yes other than never) |
| **Pre-conception Maternal health:** | | |
| Before pregnant saw a mental health professional | Questionnaire @1year, Mother self-report | Before you were pregnant, did you see a Psychiatrist, psychologist/ therapist or doctor for psychological problems for yourself? yes/no/ don’t know assumed yes. |
| History of Blood Pressure problems before pregnancy/in prior pregnancy | In maternity unit at childs birth, Medical file | Prior history: None, chronic pre-morbid high blood pressure (HBP), only in a previous pregnancy |
| History of diabetes before pregnant/in prior pregnancy | In maternity unit at childs birth, Medical file | Prior history: no, type1, type2, only in a previous pregnancy |
| During a previous pregnancy were you depressed/anxious? | In maternity unit at childs birth, maternal self report | 8249 were first-pregnancies so coded as never pregnant. No, don’t know coded as no, yes as yes |
| **Birth/delivery factors:** | | |
| Sex of child | In maternity unit at childs birth, Medical File | Male, female, unknown |
| Spontaneous labour | In maternity unit at childs birth, Medical File | Start of labour: spontaneous, induced, caesarean before start of labour |
| Mode of delivery | In maternity unit at childs birth, Medical File | Delivery: spontaneous vaginal, forceps/spatulas/vacuum, caesarean, don’t know |
| Neonatal Intensive Care Unit (NICU) | In maternity unit at childs birth, Medical File | Transfer of the child: No/Yes/don’t know – only babies needing NICU are transferred |
| Gestational age (GA) (weeks) | In maternity unit at childs birth, Medical File | Gestational age in weeks at delivery |

Fig S.1: *Frequencies of total number of pregnancy-specific-experiences (PSEs) groups.*


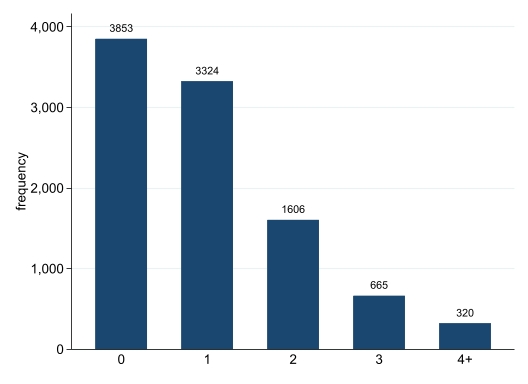


Table S.3:

*Validating ^a^Sociodemographic risk measure with financial comfort question*

| Household is: (n=9768) | No socio-demographic risk (%) | Low socio-demographic risk  (%) | Moderate socio-demographic risk (%) | High socio-demographic risk (%) |
| --- | --- | --- | --- | --- |
| Financially comfortable (1568) | 26.1 | 10.8 | 4.6 | 5.1 |
| Financially OK (4185) | 52.3 | 42.1 | 28.5 | 23.7 |
| Financially tight (3235) | 20.5 | 40.3 | 49.5 | 47.1 |
| Financially difficult (564) | 0.9 | 5.9 | 14.5 | 18.6 |
| Always in debt (106) | 0.2 | 0.8 | 2.9 | 5.5 |
| Don’t want to answer (4) | 0.02 | 0.1 | 0 | 0 |

Note: ^a^sociodemographic risk comprised of maternal age, education, relationship, migrancy and family income quintile.

| Table S.4:  *List of all sensory indicators used and their prevalence (N=9768)* | | | |
| --- | --- | --- | --- |
| At 1-year: | | Weighted prevalence for regressions | Unweighted |
| Suck fingers | Q: Does your child suck their thumb/finger?  0 – never, sometimes, don’t know  1 - Often and all of the time | 16.5% | 16.6% often |
| Easy to calm | Q: Is it easy or difficult for you to calm your child when they are upset?  0– difficult  1 - very easy, quite easy, don’t know | 8.7% | 7.3% difficult |
| Accepts confined | Q: How does your child react when placed in a confined/restricted area (eg car seat, baby seat etc?)  0– generally protests  1 - mostly accepts, sometimes accepts, don’t know | 11.0% | 11.6% protests |
| Adapts new | Q:Does your child adapt easily to new things (eg people, places, food etc.)?  0 - almost never  1- always, often, sometimes, don’t know | 16.6% | 13.4% never |
| Child anxious | Q: Have you noticed in the last few months that your child is fearful/anxious?  0-time to time, rarely/never, don’t know  1-often | 6.1% | 5.0% often |
| Unable still | Q: In the last few months, have you found that during the day your child has been particularly worked up, unable to keep still?:  0-time to time, rarely/never, don’t know  1-often | 25.1% | 23.3% often |
| Concern food | Q: are you concerned about your child’s diet?  0– no  1-yes | 2.2% | 2.1% yes |
| Problems to sleep | Q: When you put your child to bed, do they have problems going asleep (e.g. call you/cry for a long time?):  0-sometimes, never, don’t know  1-always | 16.4% | 13.7% always |
| Suck to sleep | Q: Do they need a bottle or dummy to go to sleep?  0 – no, don’t know  1 - yes | 9.6% | 7.3% |
| Accepts new food | Q: Since your child has been weaned, do they like new food?  0-often rejects it  1-Accept it easily, depends on the food, don’t know | 1.6% | 1.2% rejects |
|  | Derived from the latent class analysis in a previous study |  |  |
| Typical sensory processing group |  | 80.4% | 84.7% |
| Possible sensory difficulties group |  | 6.5% | 4.9% |
| Definite sensory difficulties group |  | 13.1% | 10.4% |

Table S.5:

Univariate associations between each candidate predictor and outcome of poor mental health compared to adequate mental health (n=9768)

| Predictor | Odds Ratio (95% Confidence Interval) | P-value | Coefficient (95% Confidence Interval) |
| --- | --- | --- | --- |
| **No sociodemographic risk (base)** |  |  |  |
| Low | 1.7 (1.2-2.2) | .001 | .51 (.22-.80) |
| Moderate | 2.6 (2.0-3.4) | .000 | .96 (.68-1.23) |
| High | 3.3 (2.0-5.7) | .000 | 1.21 (.67-1.74) |
|  |  |  |  |
| **Number pregnancy-specific-experiences (base none)** |  |  |  |
| 1 | 1.2 (0.9-1.6) | .26 | .17 (-.13-.48) |
| 2 | 1.8 (1.3 – 2.5) | .00 | .59 (.28-.89) |
| 3 | 1.8 (1.2-2.8) | .01 | .58 (.14-1.02) |
| 4+ | 2.9 (1.8-4.7) | .00 | 1.06 (.59-1.54) |
|  |  |  |  |
| **Sex (male base)** |  |  |  |
| Female | .6 (.5-.8) | .00 | -.53 (-.78- -.28) |
|  |  |  |  |
| **Pre-morbid psychological difficulties (no base)** |  |  |  |
| yes | 1.5 (1.2-1.9) | .002 | .40 (.15-.65) |
|  |  |  |  |
| **History of high blood pressure (no base)** |  |  |  |
| Yes outside a pregnancy | 3.0 (1.5-6.3) | .003 | 1.11 (.39-1.84) |
| Yes during a previous pregnancy | 1.0 (.4-2.5) | .96 | .02 (-.89-.93) |
|  |  |  |  |
| **History of Diabetes (no base)** |  |  |  |
| Yes outside a pregnancy | .7 (.3-1.7) | .45 | -.33 (-1.20-.54) |
| Yes during a previous pregnancy | 1.3 (.7-2.5) | .45 | .25 (-.40-.91) |
|  |  |  |  |
| **Mental health difficulties in a previous pregnancy (never pregnant before base)** |  |  |  |
| No mental health difficulties in previous pregnancy | .7 (.5-.9) | .004 | -.37 (-.62- -.12) |
| Yes mental health difficulties in previous pregnancy | 1.6 (1.1-2.3) | .013 | .46 (.10-.82) |
|  |  |  |  |
| **Smoked in this pregnancy** | 1.8 (1.3-2.4) | .000 | .58 (.30-.86) |
|  |  |  |  |
| **Drank alcohol in this pregnancy** | 1.0 (.8-1.4) | .76 | .04 (-.24- .32) |
|  |  |  |  |
| **How labour started (spontaneous base)** |  |  |  |
| Induced | 1.2 (.9-1.6) | .31 | .16 (-.15-.48) |
| Section (planned or emergency) | .7 (.5-1.1) | .13 | -.32 (-.73-.09) |
|  |  |  |  |
| **Mode of delivery (Normal Vaginal Delivery base)** |  |  |  |
| assisted | 1.4 (1.0-2.0) | .04 | .36 (.03-.70) |
| Planned C-section | .8 (.5-1.2) | .22 | -.29 (-.76-.17) |
| Emergency C-section | 1.4 (.9-2.0) | .11 | .30 (-.07 - .68) |
|  |  |  |  |
| **Neonatal Intensive Care Unit (NICU)** | 1.5 (.9-2.4) | .10 | .39 (-.07-.86) |
|  |  |  |  |
| **Gestational Age in weeks (GA)** | .9 (.9-1.0) | .16 | -.06 (-.14-.02) |
|  |  |  |  |
| **Sensory @ 1-year (typical base)** |  |  |  |
| Possible | 1.0 (.6-1.7) | .98 | -.01 (-.55-.54) |
| Definite | 2.7 (2.1-3.6) | .000 | 1.00 (.73-1.27) |

Figure S.2:

*Calculating risk score formula for each participant*

The log-odds (Y) can be calculated using the regression equation as follows:

Y= constant + [estimate1 x predictor1] + [estimate2 x predictor2] + [estimate n x predictor n]

The log-odds (Y) is then converted into probability (P) as follows:

P= 1/[1+ exp(-Y)]

Where P is the probability of developing the outcome and Y is the log-odds estimated using the model.

Figure S.3:

*Standard 2x2 contingency table depicting possible outcomes of a binary classification test.*

| Observed outcome | | | | |
| --- | --- | --- | --- | --- |
| Predicted group |  | Poor mental health | Adequate mental health |  |
|  | Predicted high-risk | True positive (TP) | False positive (FP) | PPV=TP/(TP+FP) |
|  | Predicted low-risk | False negative (FN) | True negative (TN) | NPV=TN/(TN+FN) |
|  |  | Sensitivity = TP/(TP+FN) | Specificity = TN/(TN+FP) |  |

Note: PPV (positive predictive value); NPV (negative predictive value).

Table S.6a:

*Comparing those included in the analysis for the prediction model (n=9768) with those excluded due to withdrawing/not participating @ 5-year follow-up (n=7081) or having missing data on some of the variables (n=1480)*

|  | Total n=18329 | Included n=9768 | Excluded n=8561 | P-value |
| --- | --- | --- | --- | --- |
| Maternal Age (Mean/SD) | 30.7 (5.04) | 30.8(5.01) | 30.6 (5.22) | .52 |
| Mother born in France (%yes) | 85.6 | 86.2 | 81.1 | <.01 |
| Maternal Education Level (%) |  |  |  | .35 |
| MSc/PhD | 33.5 | 33.6 | 33.3 |  |
| Degree | 19.0 | 19.0 | 19.5 |  |
| Leaving Cert | 39.2 | 39.5 | 36.9 |  |
| None/Junior Cert | 8.2 | 7.9 | 10.3 |  |
| Family income (%) |  |  |  | .19 |
| Top 2 quintiles | 38.0 | 38.1 | 36.7 |  |
| Middle quintile | 17.1 | 16.8 | 20.2 |  |
| Lowest 2 quintiles | 44.9 | 45.2 | 43.0 |  |
| Mother NOT in relationship (%yes) | 5.5 | 5.2 | 7.9 | .06 |
| **Cumulative Sociodemographic Risk (%)** |  |  |  | .02 |
| None | 31.4 | 31.5 | 30.7 |  |
| Low | 30.2 | 29.5 | 35.8 |  |
| Moderate | 31.4 | 32.2 | 25.5 |  |
| High | 7.0 | 6.8 | 8.1 |  |
| **Total# pregnancy-specific-experiences** (%) |  |  |  | .27 |
| 0 | 34.0 | 34.4 | 31.0 |  |
| 1 | 33.7 | 33.5 | 35.2 |  |
| 2 | 18.7 | 18.4 | 20.7 |  |
| 3 | 8.5 | 8.7 | 7.0 |  |
| 4+ | 5.1 | 5.0 | 6.1 |  |
| **Mental health problems in previous pregnancy (%)** |  |  |  | .09 |
| Never pregnant before | 29.6 | 29.2 | 35.0 |  |
| No | 62.1 | 62.4 | 57.6 |  |
| Yes | 8.3 | 8.4 | 7.4 |  |
| History of High Blood Pressure (%) |  |  |  | .49 |
| No | 97.4 | 97.3 | 98.0 |  |
| Yes | 1.2 | 1.3 | 0.6 |  |
| Only in previous pregnancy | 1.4 | 1.4 | 1.3 |  |
| History of Diabetes (%) |  |  |  | .75 |
| No | 96.4 | 96.4 | 96.3 |  |
| Yes | .96 | .98 | .73 |  |
| Only in previous pregnancy | 2.6 | 2.6 | 3.0 |  |
| History of psychological difficulties (%yes) | 23.6 | 23.1 | 27.4 | .04 |
| Sex of baby (%male) | 50.4 | 50.1 | 52.8 | .30 |
| Smoked in pregnancy (%yes) | 19.7 | 19.5 | 21.5 | .37 |
| Alcohol in pregnancy (%yes) | 24.1 | 24.0 | 24.8 | .73 |
| Start Labour (%) |  |  |  | <.001 |
| Spontaneous | 71.3 | 71.9 | 64.8 |  |
| Induced | 18.9 | 19.0 | 18.7 |  |
| Section | 9.8 | 9.1 | 16.5 |  |
| Mode of delivery (%) |  |  |  | .05 |
| Normal Vaginal Delivery | 70.5 | 71.0 | 64.2 |  |
| Assisted | 11.7 | 11.5 | 13.6 |  |
| Planned C-section | 7.4 | 7.1 | 10.7 |  |
| Emergency C-section | 10.5 | 10.4 | 11.4 |  |
| Neonatal Intensive Care Unit (NICU) (%yes) | 5.3 | 5.2 | 6.1 | .46 |
| Gestational Age (weeks) (mean/SD) | 39.19(1.46) | 39.22(1.45) | 38.91(1.47) | <.001 |

Table S.6b:

*The amount of missing data for the n=11248 who took part @5-years.*

| # variables missing | Frequency | Percent |
| --- | --- | --- |
| 0 | 9768 | 86.8 |
| 1 | 778 | 6.9 |
| 2 | 152 | 1.4 |
| 3 | 41 | 0.4 |
| 4 | 9 | 0.08 |
| 5 | 15 | 0.1 |
| 6 | 64 | 0.6 |
| 7 | 12 | 0.1 |
| 8 | 6 | 0.05 |
| 9 | 9 | 0.08 |
| 12 | 394 | 3.5 |

Table S.6c:

*The % of cases missing for each variable for the model of the n=11248 who took part at 5-years.*

| Variable | Number of observations missing | Percent |
| --- | --- | --- |
| Sociodemographic risk | 394 | 3.5 |
| Total# pregnancy specific experiences | 394 | 3.5 |
| Sex | 484 | 4.3 |
| Psychological difficulties before pregnancy | 423 | 3.8 |
| History of high blood pressure outside of pregnancy | 609 | 5.4 |
| History of diabetes outside of pregnancy | 694 | 6.2 |
| Mental health difficulties in a previous pregnancy | 774 | 6.9 |
| Smoking in current pregnancy | 500 | 4.5 |
| Alcohol use in current pregnancy | 508 | 4.5 |
| Start labour | 588 | 5.2 |
| Mode of delivery | 719 | 6.4 |
| Neonatal Intensive care unit (NICU) | 0 | 0 |
| Gestational age in weeks | 554 | 4.9 |
|  |  |  |
| Sensory group for 1-year model | 394 | 3.5 |

Figure S.4:

*Flow-diagram of participants*

N=18329 babies recruited

N=11248 took part at 5-years and thus had outcome

N=9768 no missing data

Table S.7:

*Model performance statistics for the model after internal validation using bootstrapping.*

| Model: | Performance | | |
| --- | --- | --- | --- |
| Statistic | Original apparent | Optimism | Optimism adjusted |
| C-statistic | 0.67 (0.64-0.69) | -.01 | 0.66 (0.57-0.71) |
| CITL | 0 (-0.03) | -.004 | -.02 |
| C-slope | 1 (1.04) | -.02 | 1.02 |

Note. Slope >1 = under fitted. CITL <0 = over predicting. There was minimal optimism adjustment to the C-statistic indicating good internal performance in terms of discrimination. The mis-calibration in CITL is very small at <1% basically zero miscalibration in overall risks. The calibration slope suggests a moderate amount of shrinkage is required to adjust the predictor effects in the model ie under fitted but over predicting.

Fig S.5a: *Calibration instability plot*


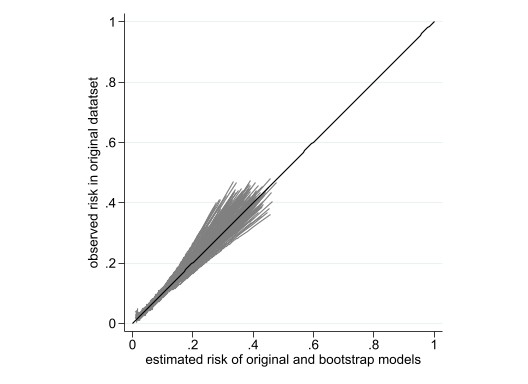


Note. The bootstrapped curves are overlaid on this plot with the original calibration curve (the wider the spread of the curves, the greater the instability concern).

Fig S.5b: *Classification instability plot*


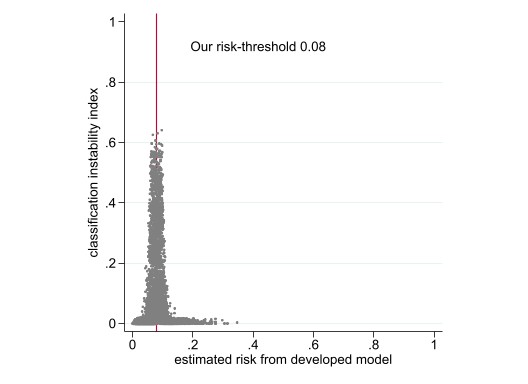


Note. Each individuals classification index (y-axis) from bootstrapped models is plotted against their original predicted value (X-axis). A narrow distribution with values close to zero for most people except those close to the threshold indicates stable classifications.

Fig S.5c:

*C-statistic histogram*


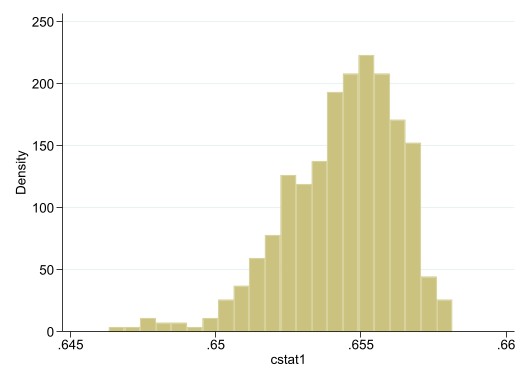


Note. In terms of discrimination, the C-statistic histogram shows very little variability in the C-statistic estimates across bootstrap samples, again showing good stability.

Fig S.5d:

*Mean Absolute prediction error (MAPE) plot*


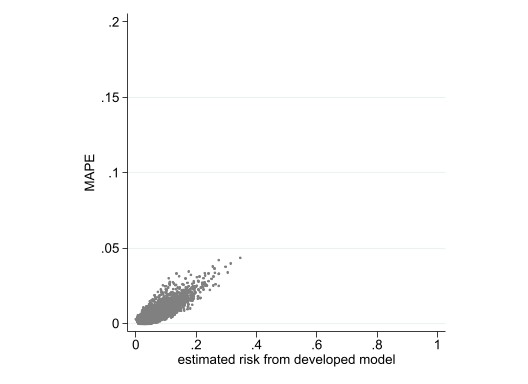


Note. The average MAPE is 0.006 indicating very low instability in each individual’s prediction between the original model and the bootstrapped models on average.

Fig S.6:

*Accuracy of classification at ≥8% risk-threshold.*


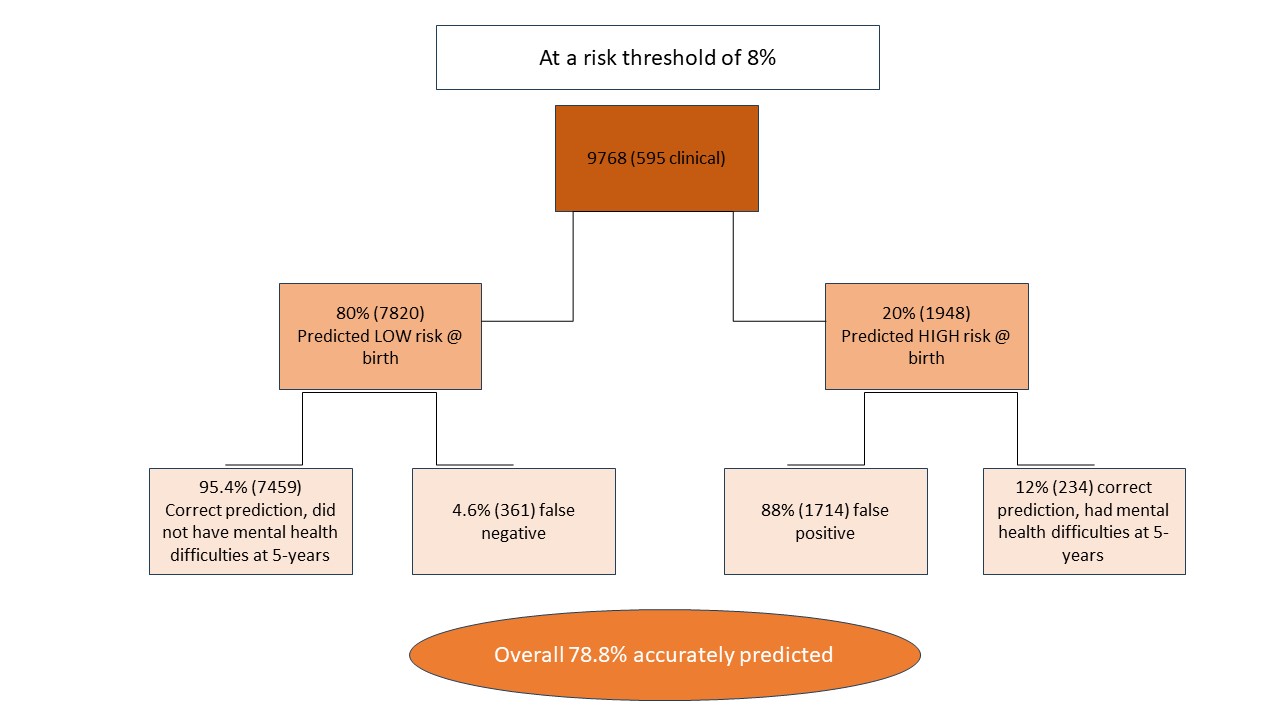


Table S.8:

*The proportion of children with poor mental health at 5-years examined across sex, social risk and Neonatal Intensive Care Unit (NICU)*

|  | % poor mental health (95%CI) |
| --- | --- |
| **Sex:** |  |
| Male (n=4940) | .08 (.07-.08) |
| Female (n=4828) | .05 (.04-.05) |
| **Cumulative sociodemographic risk:** |  |
| None (n=4406) | .04 (.04-.05) |
| Low (n=3064) | .06 (.05-.07) |
| Moderate (n=2023) | .09 (.08-.10) |
| High (n=275) | .13 (.09-.18) |
| **NICU:** |  |
| No NICU (n=8014) | .06 (.06-.07) |
| Yes NICU (n=477) NB only ≥33weeks gestation | .07 (.05-.10) |

Table S.9:

Comparing the model performance in our different subgroups of interest (sex, sociodemographic risk and Neonatal Intensive Care Unit (NICU)).

| Group | N= | ROC area | Standard Error | 95% CI |
| --- | --- | --- | --- | --- |
| All | 9768 | 0.67 | 0.01 | 0.64-0.69 |
| **Sex** |  |  |  |  |
| Male | 4940 | 0.64 | 0.02 | 0.61-0.67 |
| Female | 4828 | 0.66 | 0.02 | 0.62-0.70 |
| **Sociodemographic Risk** |  |  |  |  |
| None | 4406 | 0.65 | 0.02 | 0.61-0.69 |
| Low | 3064 | 0.63 | 0.02 | 0.59-0.67 |
| Moderate | 2023 | 0.63 | 0.02 | 0.59-0.67 |
| High | 275 | 0.65 | 0.05 | 0.54-0.75 |
| **NICU** |  |  |  |  |
| No | 9291 | 0.66 | 0.01 | 0.64-0.68 |
| Yes | 477 | 0.78 | 0.04 | 0.70-0.87 |

Table S.10:

*Co-efficient values for the sensory variable and model performance of the sensory model at 1-year when included as a possible candidate predictor and later start-point.*

| **Predictors** | **1-year model** |
| --- | --- |
|  | **Coefficient*** |
| Intercept | -2.92 |
| Total number of pregnancy-specific adverse experiences: | |
| 0 | Ref |
| 1 | .05 |
| 2 | .46 |
| 3 | .48 |
| 4+ | 1.12 |
| Cumulative sociodemographic risk: | |
| None | Ref |
| Low | .12 |
| Moderate | .53 |
| High | .56 |
| History of pre-morbid blood pressure: | |
| No | Ref |
| Yes | .004 |
| History of psychological difficulties before being pregnant: | |
| Yes | Ref |
| No | -0.10 |
| Mental health difficulties in a previous pregnancy: | |
| Yes | Ref |
| No | -0.38 |
| Never pregnant before | .006 |
| Smoked during the pregnancy: | |
| Yes | Ref |
| No | -0.30 |
| Start of labour: | |
| C-section (plan/emergency) | Ref |
| Spontaneous | -0.02 |
| Induced | 0.04 |
| Infant sex: | |
| Female | Ref |
| Male | 0.38 |
| Sensory profile @1-year: | |
| Typical | Ref |
| Possible | -.06 |
| Definite | 0.87 |
| **Discrimination & calibration performance metrics:** | |
| Area-Under-the-Curve (AUC) | 0.69 (0.67-0.71) |
| Calibration slope | 1.05 |
| Expected/Observed (E/O) | 1.02 |
| Calibration-in-the-large (CITL) | -0.02 |
| Brier Score | 0.06 |

*coefficient from LASSO ie includes shrinkage
